# Supplementary material for: The causal relationship between the human gut microbiota and pyogenic arthritis: a Mendelian randomization study
Source: Front Cell Infect Microbiol. 2024 Nov 26;14:1452480. doi: 10.3389/fcimb.2024.1452480 (PMC11629706; doi:10.3389/fcimb.2024.1452480)
Supplement: Supplementary file 1 [file DataSheet1.zip › mendelian test/ebi-a-GCST90027564.csv_forest.pdf]

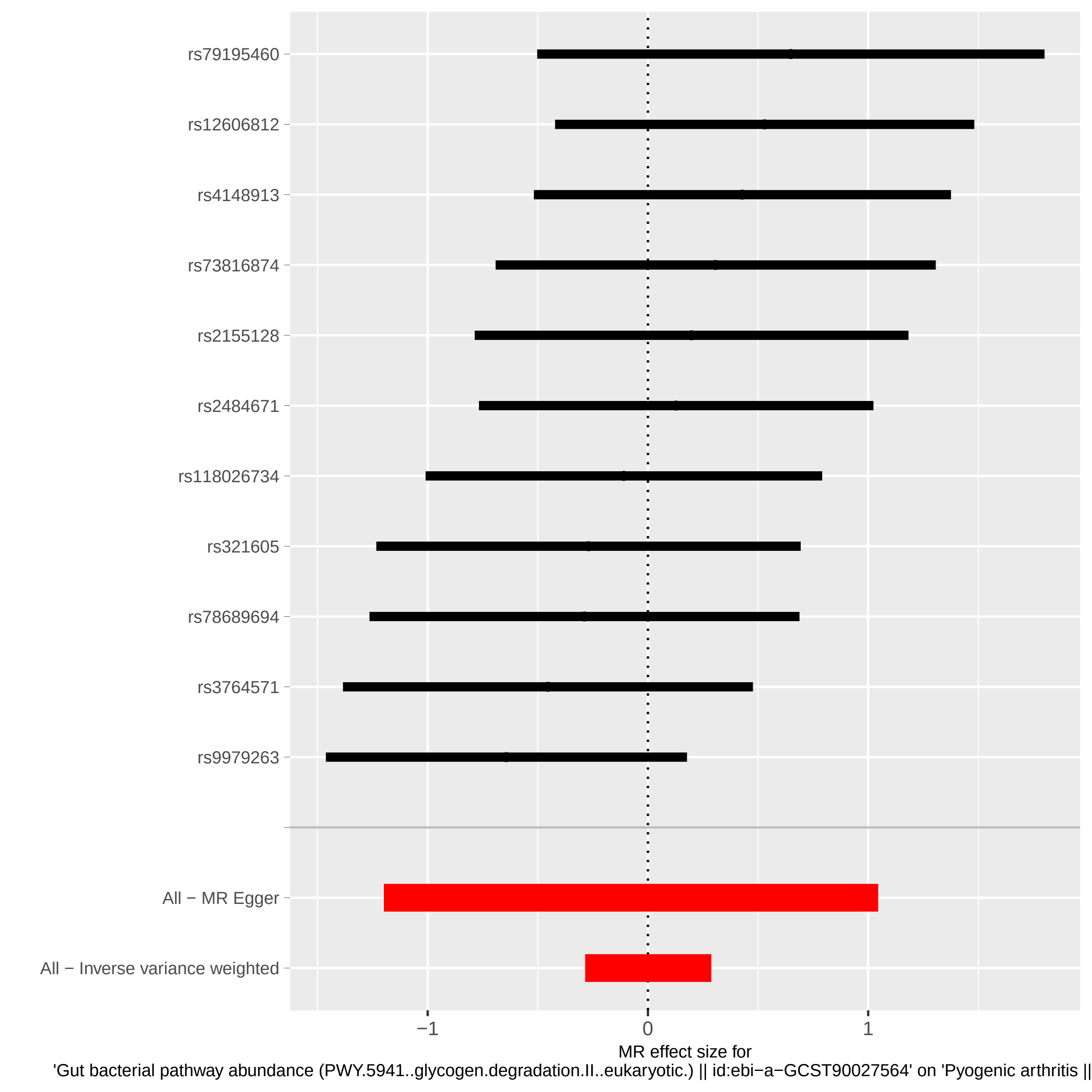

rs79195460
rs12606812
rs4148913
rs73816874
rs2155128
rs2484671
rs118026734
rs321605
rs78689694
rs3764571
rs9979263
All − MR Egger
All − Inverse variance weighted
−1
0
1
MR effect size for
'Gut bacterial pathway abundance (PWY.5941..glycogen.degradation.II..eukaryotic.) || id:ebi−a−GCST90027564' on 'Pyogenic arthritis ||
